# Mucin Muc2 Deficiency and Weaning Influences the Expression of the Innate Defense Genes Reg3β, Reg3γ and Angiogenin-4

**DOI:** 10.1371/journal.pone.0038798

**Published:** 2012-06-19

**Authors:** Nanda Burger-van Paassen, Linda M. P. Loonen, Janneke Witte-Bouma, Anita M. Korteland-van Male, Adrianus C. J. M. de Bruijn, Maria van der Sluis, Peng Lu, Johannes B. Van Goudoever, Jerry M. Wells, Jan Dekker, Isabelle Van Seuningen, Ingrid B. Renes

**Affiliations:** 1 Laboratory of Pediatrics, Division of Neonatology, Erasmus MC-Sophia, Rotterdam, the Netherlands; 2 Host-Microbe-Interactomics Group, Wageningen University, Wageningen, the Netherlands; 3 Top Institute Food and Nutrition, Wageningen, the Netherlands; 4 Animal Sciences Department, Wageningen UR, the Netherlands; 5 Inserm, U837, Jean-Pierre Aubert Research Center, Team 5 « Mucins, epithelial differentiation and carcinogenesis », Lille, France; Charité-University Medicine Berlin, Germany

## Abstract

**Background:**

Mucin Muc2 is the structural component of the intestinal mucus layer. Absence of Muc2 leads to loss of this layer allowing direct bacterial-epithelial interactions. We hypothesized that absence of the mucus layer leads to increased expression of innate defense peptides. Specifically, we aimed to study the consequence of Muc2 deficiency (Muc2^−/−^) on the expression of regenerating islet-derived protein 3 beta (Reg3β), regenerating islet-derived protein 3 gamma (Reg3γ), and angiogenin-4 (Ang4) in the intestine shortly before and after weaning.

**Methods:**

Intestinal tissues of Muc2^−/−^ and wild-type (WT) mice were collected at postnatal day 14 (P14, i.e. pre-weaning) and P28 (i.e. post-weaning). Reg3β, Reg3γ, and Ang4 expression was studied by quantitative real-time PCR, Western-blot, *in situ* hybridization, and immunohistochemistry.

**Results:**

Reg3β and Reg3γ were expressed by diverging epithelial cell types; namely enterocytes, Paneth cells, and goblet cells. Additionally, Ang4 expression was confined to Paneth cells and goblet cells. Expression of *Reg3β*, *Reg3γ*, and *Ang4* differed between WT and Muc2^−/−^ mice before and after weaning. Interestingly, absence of Muc2 strongly increased *Reg3β* and *Reg3γ* expression in the small intestine and colon. Finally, morphological signs of colitis were only observed in the distal colon of Muc2^−/−^ mice at P28, where and when expression levels of *Reg3β*, *Reg3γ*, and *Ang4* were the lowest.

**Conclusions:**

Expression of Reg3 proteins and Ang4 by goblet cells point to an important role for goblet cells in innate defense. Absence of Muc2 results in up-regulation of *Reg3β* and *Reg3γ* expression, suggesting altered bacterial-epithelial signaling and an innate defense response in Muc2^−/−^ mice. The inverse correlation between colitis development and *Reg3β*, *Reg3γ*, and *Ang4* expression levels might point toward a role for these innate defense peptides in regulating intestinal inflammation.

## Introduction

The mucus layer that covers the intestinal epithelium forms a physical barrier against bacteria and is thereby an important component of the innate defense. The mucin MUC2 is the structural component of the colonic mucus layer. Interestingly, particular bacteria can use the glycan-chains of mucins as a nutrient source or bind to the mucins as a foothold for colonization. Previously, it has been demonstrated that the intestinal mucus layer exists of two separate layers [1], [2]. The inner layer is densely packed, firmly attached to the epithelium, and devoid of bacteria. In contrast, the outer layer is colonized by bacteria, and has a less firm structure. Breaches in this protective mucus layer allow for direct contact between bacteria and the epithelial cells [3], which leads to an inflammatory response. Muc2^−/−^ mice lacking the mucus layer develop colitis spontaneously [1], [4].

In human intestinal inflammatory diseases such as ulcerative colitis and necrotizing enterocolitis (NEC), the synthesis of MUC2 mucin is decreased [5], [6], [7], [8], [9], which might lead to increased bacterial-epithelial interaction. Bacteria are known to play a key role in the development of colitis as the development of colitis in genetically engineered rodent models of inflammatory bowel diseases (IBD) such as Il-10 deficient mice and HLA-B27 transgenic rats is not observed when these animals are maintained under germ-free conditions [10], [11], [12]. However, colonization of Il-10 deficient mice and HLA-B27 transgenic rats with normal enteric microbiota leads to severe and chronic colitis. IBD and NEC are not caused by specific intestinal bacterial species, but altered microbial profiles might be involved in the pathogenesis of these diseases. In the pathophysiology of IBD and NEC, a dysbiosis of the microbiota is strongly implicated [13], [14].

The composition of the microbiota is shaped in part by specific epithelial proteins, *e.g.* defensins and antimicrobial C-type lectins [15]. Some antimicrobial proteins, such as most α-defensins, are expressed constitutively and do not require bacterial signals for their expression [16]. However, expression of a subset of bactericidal proteins is, at least partly, controlled by recognition of microbe associated molecular patterns by pattern recognition receptors expressed by the epithelial cells [17]. For example, expression of the antimicrobial C-type lectin regenerating islet-derived protein 3 gamma (Reg3γ, also called HIP/PAP in humans), is up-regulated in the small intestine and colon after bacterial reconstitution of germ-free mice [18], [19]. It has recently been demonstrated that Reg3γ restricts bacterial colonization of the intestinal epithelial surface and consequently limits activation of adaptive immune responses by the microbiota [20]. In this respect, the mucus layer is of great importance as it functions as a mesh that retains bactericidal proteins such as Reg3γ, and also molecules with immunologic properties such as sIgA [21]. Interestingly, Reg3γ^−/−^ mice exhibited a marked increase in numbers of mucosa associated Gram-positive bacteria [20]. Expression of the bactericidal peptide angiogenin-4 (Ang4), the orthologue of human ANG, is induced upon colonization with *Bacteroides thetaiotaomicron*, an anaerobe Gram-negative microbe that belongs to the normal mouse and human microbiota [22]. Furthermore, in conventionally raised mice the expression of Reg3γ and Ang4 increases substantially after weaning [19], [22], when the complexity of the microbiota increases, and during experimental intestinal infection [23], [24], [25]. Regenerating islet-derived protein 3 beta (Reg3β), Reg3γ and HIP/PAP appear to be important in inflammatory diseases and intestinal injury as their expression is increased in IBD patients and in dextran sulfate sodium models of mouse colitis [18]. Finally, Reg3β^−/−^ mice show impaired clearance of Gram-negative bacteria *Yersinia pseudotuberculosis* [26] and *Salmonella enteritidis* [27]. Yet, both unchallenged Reg3β^−/−^ and Reg3γ^−/−^ mice do not show gross morphological changes in the intestines.

The aim of this study was to investigate the effect of the mucus layer on Reg3β, Reg3γ, and Ang4 expression and localization in the small intestine and colon using the Muc2^−/−^ mouse as a model. With this approach we aimed to i) study the consequence of Muc2 deficiency, i.e. absence of a protective mucus layer and, ii) analyze the effect of weaning (i.e., transfer from breast milk to pelleted food), when the density and complexity of the microbiota increases significantly. This study demonstrates that the expression of the innate defense genes *Reg3β*, *Reg3γ*, and *Ang4* differed between wild-type (WT) and Muc2^−/−^ mice before and after weaning. Additionally, it highlights a new role for goblet cells in host innate immunity by demonstrating that they can produce the bactericidal peptides Reg3β, Reg3γ, and Ang4.

## Methods

### Animals

Muc2^−/−^ mice were bred as previously described [4]. All mice were housed in the same specific pathogen-free environment with free access to standard rodent pellets (Special Diets Services, Witham, Essex, England) and acidified tap water in a 12-hour light/dark cycle. All animal experiments were reviewed by and performed with approval of the Erasmus MC Animal Ethics Committee (approval number: EUR 1074), Rotterdam, the Netherlands. WT and Muc2^−/−^ mice were tested negative for *Helicobacter hepaticus* and norovirus infection.

### Experimental Setup

Wild-type (WT) and Muc2^−/−^ littermates were housed together with their birth mothers until weaning at the age of 21 days. After weaning, male WT and Muc2^−/−^ mice remained housed with their littermates. Male WT and Muc2^−/−^ mice were sacrificed at the postnatal ages of 14 days (P14) and 28 days (P28). Intestinal tissues were excised and either fixed in 4% (w/v) paraformaldehyde in phosphate-buffered saline (PBS), stored in RNAlater® (Sigma-Aldrich Chemie, Zwijndrecht, the Netherlands) at −20°C, or frozen in liquid nitrogen and stored at −80°C.

### Quantitative Real-time PCR

Total RNA was prepared using the RNeasy midi-kit (Qiagen, Venlo, the Netherlands). Total RNA (1.5 µg) was used to prepare cDNA using a standard protocol. The mRNA expression levels of *Reg3β*, *Reg3γ*, *Ang4* and Lysozyme type P (*lysozyme-P*), as well as the ‘housekeeping’ gene β-Actin (*Actb*) were quantified using real-time PCR analysis based upon the intercalation of SYBR*^®^* Green on an ABI prism 7900 HT Fast Real Time PCR system (PE Applied Biosystems) as previously described [4]. All primer combinations were designed using OLIGO 6.22 software (Molecular Biology Insights) and purchased from Invitrogen. An overview of all primer sequences is given in Table 1.

**Table 1:** Primer sequences for quantitative real-time PCR.

Gene	Forward primer	Reverse primer
***Reg3β***	TGG GAA TGG AGT AACAAT G	GGC AAC TTC ACC TCACAT
***Reg3γ***	CCA TCT TCA CGT AGCAGC	CAA GAT GTC CTG AGGGC
***Ang4***	TTG GCT TGG CAT CATAGT	CCA GCT TTG GAA TCACTG

### Western-blot Analysis

Jejunal and distal colonic samples were homogenized in 500 µl HIS buffer (50 mM Tris/HCl pH 7.5, 5 mM EDTA pH 8.0, 1% Triton X-100, 10 mM iodacetamide, 100 µg/ml soy bean trypsin inhibitor, 10 µg/ml pepstatine A, 10 µg/ml leupeptin, 1% (w/v) aprotinin and 1 mM PMSF). Total protein concentration was quantified using the bicinchoninic acid assay (Pierce assay, Perbio Science, Etten-Leur, the Netherlands). Twenty µg of total protein was denatured at 95°C for 5 min in Laemmli loading buffer and subjected to 12% (w/v) SDS-polyacrylamide gel electrophoresis. The separated proteins were transferred to nitrocellulose membranes (Protan BA 83, 0.2 µm) and the blots were blocked for 1 h at room temperature in 5% (w/v) non-fat dry milk (Campina Melkunie, Eindhoven, the Netherlands) dissolved in phosphate-buffered saline containing 0.1% (v/v) Tween-20 (PBST). Blots were incubated overnight at 4°C with anti-β-Actin antibody (1∶10,000 in PBST, Abcam, ab6276) or with the custom made primary antibodies against Reg3β and Reg3γ (1∶20,000 in PBST, Eurogentec, Seraing, Belgium). These antibodies were generated in rabbits against the synthetically produced peptides, using the peptide sequences GEDSLKNIPSARISC (Reg3β) and MIKSSGNSGQYVC (Reg3γ). The chosen peptide sequences correspond to unique sequences within the respective Reg3 proteins, and allow for differentiation between the Reg3β and Reg3γ proteins. Serum from immunized rabbits was affinity purified using the respective peptides. Selectivity and cross reactivity of the generated antibodies for the Reg3 proteins were checked by ELISA. Finally, blots were incubated with the secondary antibody goat-anti-rabbit IRDye® 800CW (1∶20,000, Li-cor, Westburg, Leusden, the Netherlands) for Reg3β and Reg3γ, and goat-anti-mouse IRDye®680CW (1∶20,000, Li-cor) for β-Actin. Signals were detected with the Odyssey scanner (Li-cor). Serial dilution series of the protein samples were analyzed to ensure that the quantification of each protein by its cognate antibody was performed in the linear range of this technique. Expression of each protein is expressed relative to the expression of β-Actin.

### Histology

Tissue fixed in 4% (w/v) paraformaldehyde in PBS was prepared for light microscopy, and 4-µm-thick sections were stained with hematoxylin and eosin (H&E) and periodic acid Schiff’s (PAS) staining to study morphological changes and detect goblet cells, respectively. To detect differences in mucosal thickness in the colon, 10 well-oriented crypts were chosen per intestinal segment and measured using calibrated Leica Application Suite software, version 3.2.0 (Leica Microsystems BV, Rijswijk, the Netherlands).

### Immunohistochemistry

Four-micrometer-thick sections were prepared for immunohistochemistry as described previously [28] using the Vectastain Elite ABC kit (Vector Laboratories, Burlingame, CA) and the staining reagent 3,3′-diaminobenzidine. The antigens were unmasked by heating the sections for 20 min in 0.01 M Tris/HCl (pH 9.0) supplemented with 0.05% (v/v) EGTA at 100°C. Expression of Reg3β was detected using a commercial anti-mouse Reg3β antibody (1∶500 diluted in PBS containing 1% bovine serum albumin and 0.1% Triton X-100, R&D Systems Europe Ltd., Abingdon, United Kingdom, AF5110) and with the above described custom-made antibody against Reg3β (1∶25,000 in PBS). Reg3γ was detected using the above described custom made antibody against Reg3γ (1∶25,000 in PBS). Expression of angiogenin-4 was detected using an anti-human-angiogenin antibody (1∶50 diluted in PBS containing 1% bovine serum albumin and 0.1% Triton X-100, R&D Systems Europe Ltd., Abingdon, United Kingdom, AF265). To identify goblet cells HA1 antibody, which is specific for Muc4 was used as described previously [29]. As the commercial and the custom-made Reg3β antibody gave similar staining patterns only data obtained with the commercial Reg3β antibody are shown.

### Probe Preparation

Digoxigenin-11-UTP-labelled RNA probes were prepared according to the manufacturer’s instructions (Boehringer Mannheim GmbH, Biochemica, Mannheim, Germany) using T3 and T7 RNA polymerase. Gene fragments of *Ang4*, *Reg3β* and *Reg3γ* were amplified, using the primers listed in Table 2, and cloned in pBluescript II SK.

**Table 2:** Primer sequences for probe preparation for ISH.

Gene	Forward primer	Reverse primer	Amplified product (bp)
***Reg3β***	TGG GAA TGG AGT AAC AAT G	ATG TGA GGT GAA GTT GCC	146
***Reg3γ***	CAA TCA CTG TGG TAC CCT G	GAT TTT CTC CTT CTC TGG C	229
***Ang4***	CCA GCT TTG GAA TCA CTG T	CTA TGA TGC CAA GCC AA	151

### *In situ* Hybridization (ISH)

Non-radioactive ISH was performed according to a previously described method [30]. The digoxigenin-labeled hybrids were detected by incubation with anti-digoxigenin (Fab, 1∶1000 in TBS/1% BA + 1% v/v sheep serum, Roche) conjugated to alkaline phosphatase for 2.5 h at room temperature. Thereafter, sections were washed in 0.025% (v/v) Tween in Tris-buffered saline (pH 7.5). For staining, sections were layered with detection buffer (0.1 M Tris/HCl, 0.1 M NaCl, 0.05 M MgCl_2_, pH 9.5) containing 0.33 mg/ml 4-nitroblue tetrazolium chloride, 0.16 mg/ml 5-bromo-4-chloro-3-indolyl-phosphate, 8% (v/v) polyvinyl alcohol (*Mw* 31,000–50,000, Aldrich Chemical, Milwaukee, WI, USA), and 1 mM levamisole (Sigma). The color reaction was performed overnight in the dark and was stopped when the desired intensity of the resulting blue precipitate was reached. Finally, sections were washed in 10 mM Tris/HCl pH 9.5 containing 1 mM EDTA, washed in distilled water, and mounted with Aquamount improved (Gurr, Brunschwig, Amsterdam, the Netherlands).

### Statistical Analysis

All data are expressed as median. Statistical significance was assessed using the Mann-Whitney U test (Prism, version 5.00; GraphPad software, San Diego, CA). The data were considered statistically significant at *P<*0.05.

## Results

### Clinical Symptoms and Intestinal Morphology

**Figure 1:**
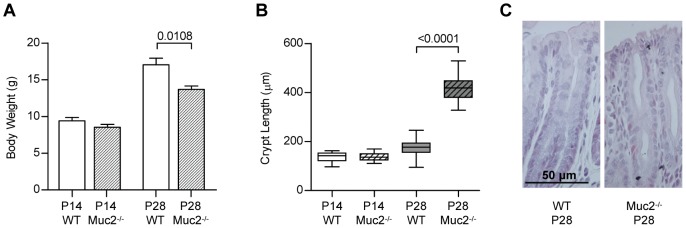
Clinical symptoms and intestinal morphology. Body weights (A) and crypt lengths in the distal colon (B) of WT and Muc2^−/−^ mice at P14 and P28. Crypth length values are depicted as box-and-whiskers diagrams (maximum value, upper quartile, median, lower quartile and minimal value, respectively) *P* values are indicated when body weights/ crypt lengths differ statistically between groups. (C) H&E staining of distal colonic tissue of Muc2^−/−^ mice at P28.

At P14, when the mice received breast milk, there were no significant differences in body weights between Muc2^−/−^ and WT mice (Figure 1A). However, at P28, when mice had been transferred from breast milk to solid food, the body weights of Muc2^−/−^ mice were significantly lower than that of WT mice (*P* = 0.0108). Clinical signs of colitis like rectal bleeding, bloody stools or rectal prolapse were not observed in Muc2^−/−^ mice at P14 nor at P28. Morphological signs of colitis were only observed in the distal colon of Muc2^−/−^ mice at P28, but not in the distal colon at P14, neither in the proximal colon and small intestine at P14 or P28 (*Figure S1*). More specifically, at P28 the distal colonic tissue from Muc2^−/−^ mice showed increased crypt lengths, (Figure 1B and *Figure S1K and S1L*) and flattening of the epithelial cells (Figure 1C).

### Localization of Reg3β, Reg3γ, and Ang4 mRNAs and Proteins in the Small Intestine

**Figure 2:**
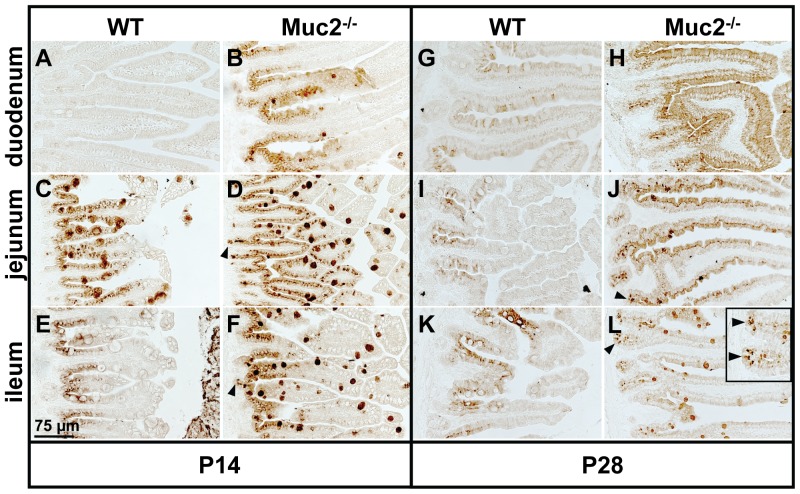
Localization of Reg3β protein in the small intestine. Immunohistochemical staining of Reg3β in the duodenum, jejunum, and ileum of WT (A, C, E, G, I, K) and Muc2^−/−^ mice (B, D, F, H, J, L) at P14 (A–F) and P28 (G–L). Bar represents 75 µm. Arrowheads indicate Reg3β-positive Paneth cells.

**Figure 3:**
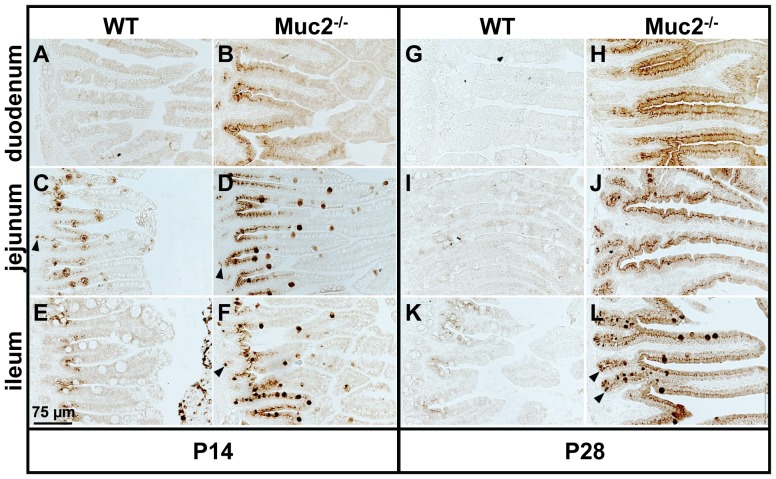
Localization of Reg3γ protein in the small intestine. Immunohistochemical staining of Reg3γ in the duodenum, jejunum, and ileum of WT (A, C, E, G, I, K) and Muc2^−/−^ mice (B, D, F, H, J, L) at P14 (A–F) and P28 (G–L). Bar represents 75 µm. Arrowheads indicate Reg3γ-positive Paneth cells.

We first determined the expression pattern of the Reg3 proteins in the small intestine of WT and Muc2^−/−^ mice by immunohistochemistry. Interestingly, we did find differences in the localization of the Reg3 proteins, although there were no major differences in small intestinal morphology between WT and Muc2^−/−^ mice. Specifically, Reg3β and Reg3γ were not expressed in the duodenum of WT mice at P14 (Figure 2 and 3). However, the jejunum and ileum of WT mice clearly express Reg3β and Reg3γ at this time point. At P28 Reg3γ was still expressed in the jejunum and ileum of WT mice, but the expression was weak. Reg3β expression at P28 was also weak but present in each region of the small intestine of WT mice. In sharp contrast, in Muc2^−/−^ mice the Reg3 proteins were strongly expressed in the entire small intestine from duodenum till ileum (Figure 2 and 3) at P14 as well as P28.

**Figure 4:**
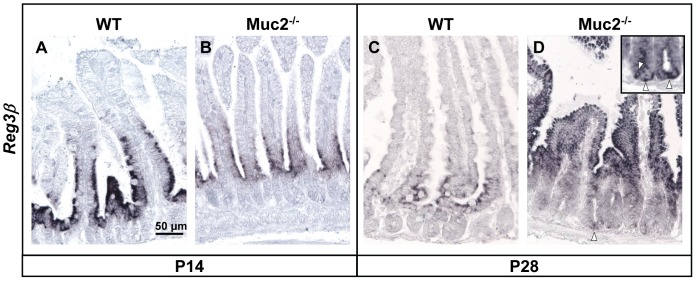
Localization of *Reg3β* mRNA in the jejunum. ISH for *Reg3β* mRNA in the jejunum of WT and Muc2^−/−^ mice at P14 (A & B) and P28 (C & D) (arrowheads in insert in panel D indicate *Reg3β*mRNA-positive cells at the bottom of the crypts in Muc2^−/−^ at P28). Bar represents 50 µm.

To determine the expression pattern of Reg3 mRNAs and proteins in more detail we focused on the jejunum. In both, WT mice and Muc2^−/−^ mice, *Reg3β* mRNA was detected by ISH and observed in epithelial cells at the base of the villi at P14 (Figure 4A and B). In contrast, at P28 the expression pattern of *Reg3β* mRNA remained limited to the villus base in WT mice, but had extended to the upper part of the villi in Muc2^−/−^ mice (Figure 4D). Interestingly, in Muc2^−/−^ mice *Reg3β* mRNA was also observed in epithelial cells at the base of the crypts (Figure 4D). *Reg3γ* mRNA was observed in epithelial cells at the crypt bottom till the tips of the villi in WT and Muc2^−/−^ mice at P14 and P28 (data not shown).

Immunohistochemical analysis revealed that in WT as well as Muc2^−/−^ mice enterocytes were Reg3β- and Reg3γ-positive (P14 and P28) (Figure 2 and 3). Moreover, in WT mice at P14 and in Muc2^−/−^ mice at P14 and P28, Reg3β- and Reg3γ-positive cells had a ‘bell/goblet’ shape suggesting that these cells could be goblet cells. Staining of serial section of the jejunum with the goblet cell marker Muc4 [29] or PAS demonstrated that the bell/goblet-shaped Reg3β- and Reg3γ-positive cells in WT and Muc2^−/−^ mice were indeed goblet cells (*Figure S2*). Furthermore, in WT mice at P14 as well as in Muc2^−/−^ mice at P14 and P28 Paneth cells at the bottom of the crypts were Reg3γ-positive (Figure 3). Yet, Reg3β-positive Paneth cells at the bottom of the crypts were only observed in Muc2^−/−^ mice, but not in WT mice (Figure 2).

**Figure 5:**
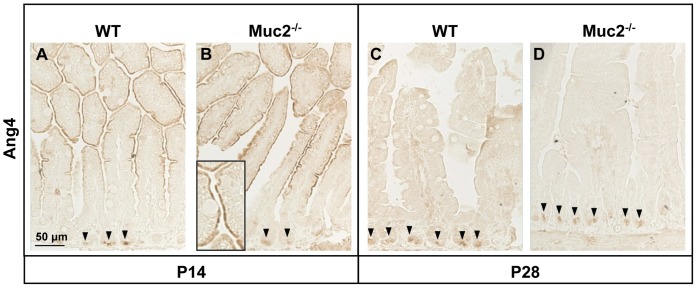
Localization of Ang4 protein in the jejunum. Localization of Ang4 in the jejunum of WT and Muc2^−/−^ mice at P14 (A & B) and P28 (C & D). Arrowheads indicate Ang4-positive Paneth cells, and the insert in panel B shows enterocyte-specific Ang4 expression at P14. Bar represents 50 µm.

Finally, small intestinal *Ang4* mRNA was localized in Paneth cells of both WT and Muc2^−/−^ mice at both ages studied (*Figure S3,* showing *Ang4* mRNA in Muc2^−/−^ mice at P14). Yet, at P14, *Ang4* mRNA was not only observed in the Paneth cells but also at the apical side of the villus enterocytes in both types of mice. Ang4 protein expression patterns were similar to the *Ang4* mRNA expression patterns with expression localized to Paneth cells at P14 and P28, and to enterocytes along the villi at P14 in both WT and Muc2^−/−^ mice (Figure 5). Moreover, the abundance of Ang4-positive Paneth cells increased from P14 to P28 in both types of mice.

### Localization of Reg3β, Reg3γ, and Ang4 Proteins in the Colon

**Figure 6:**
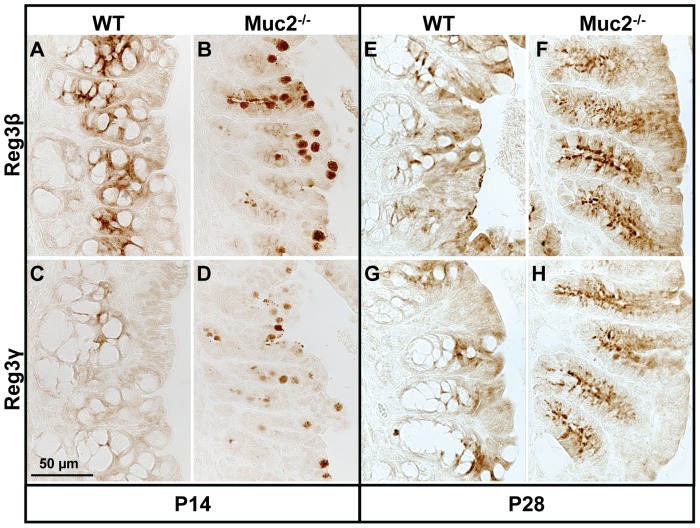
Localization of Reg3β protein in the proximal colon. Reg3β (A, B, E, F) and Reg3γ (C, D, G, H) expression in the proximal colon at P14 (A–D) and P28 (E–H). Note, in WT mice Reg3β and Reg3γ were only expressed by enterocytes, whereas in Muc2^−/−^ mice these proteins were expressed by enterocytes and putative ‘goblet’ cells. Bar represents 50 µm.

We also determined the expression pattern of the Reg3 and Ang4 proteins in the colon of WT and Muc2^−/−^ mice by immunohistochemistry. Both Reg3β and Reg3γ were undetectable in the distal colon of WT and Muc2^−/−^ mice at P14 or P28 (data not shown). However, in the proximal colon of WT and Muc2^−/−^ mice these Reg3 proteins were expressed within the epithelial cells in the crypt epithelium as well as the surface epithelium at both time points investigated (Figure 6). Interestingly, in the proximal colon of WT mice Reg3β and Reg3γ were only expressed by enterocytes, whereas in Muc2^−/−^ mice these proteins were expressed by enterocytes as well as putatively by goblet cells, as based on the ‘goblet’ shape of the Reg3β- and Reg3γ-positive cells in the Muc2^−/−^ mice. Analysis of serial sections stained with the goblet cell marker Muc4 or with PAS revealed that these Reg3β- and Reg3γ-expressing cells with a goblet shape were indeed goblet cells (data not shown). The abundance of cells with the typical bell/goblet shape seemed to decrease progressively from P14 to P28 in the proximal colon of the Muc2^−/−^ mice.

**Figure 7:**
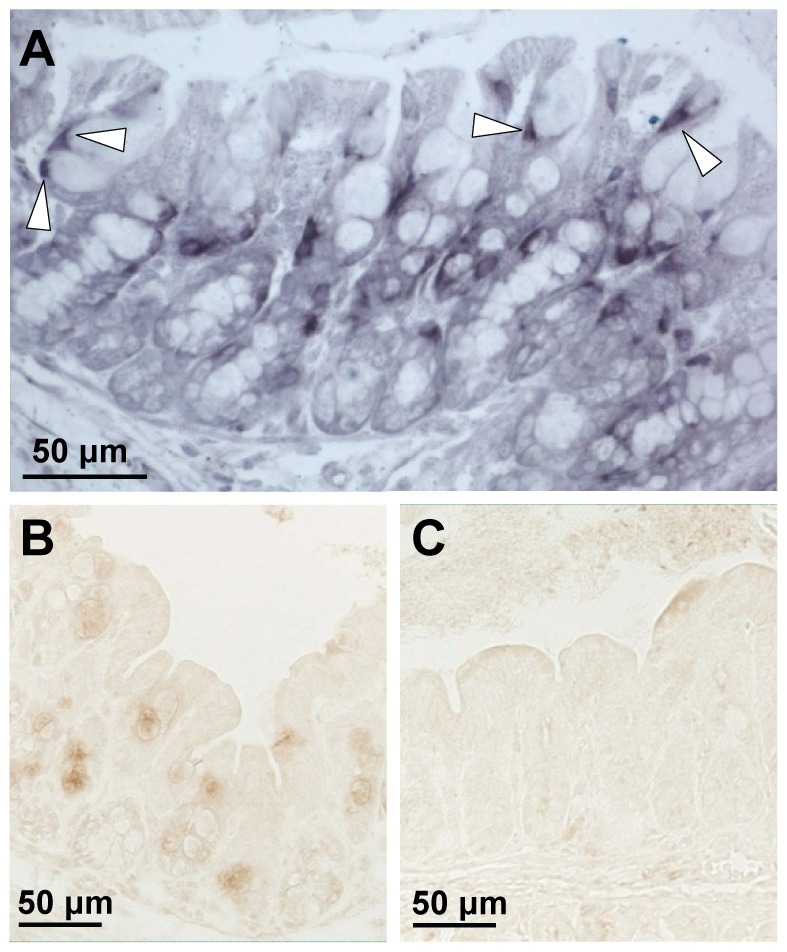
Goblet cell-specific expression of *Ang4* mRNA and protein in the proximal colon. Localization of *Ang4* mRNA in goblet cells of WT mice by ISH (A) at P28. Localization of Ang4 protein by immunohistochemistry in WT (B) and Muc2^−/−^ (C) mice at P28. Arrows indicate *Ang4* mRNA in goblet cells. Bar represents 50 µm.

Ang4 was hardly detectable in the distal colon of WT and Muc2^−/−^ mice (data not shown). However, in the proximal colon Ang4 was clearly expressed within the crypts and surface epithelium of both WT and Muc2^−/−^ mice at P14 and only in WT mice at P28, where it was expressed by goblet cells (Figure 7B and 7C). Detection of *Ang4* mRNA by ISH demonstrated that Ang4 was indeed expressed by goblet cells in the proximal colon of WT mice (Figure 7A).

### Quantitative Analysis of Reg3β, Reg3γ, Ang4, and Lysozyme-P mRNA Levels

**Figure 8:**
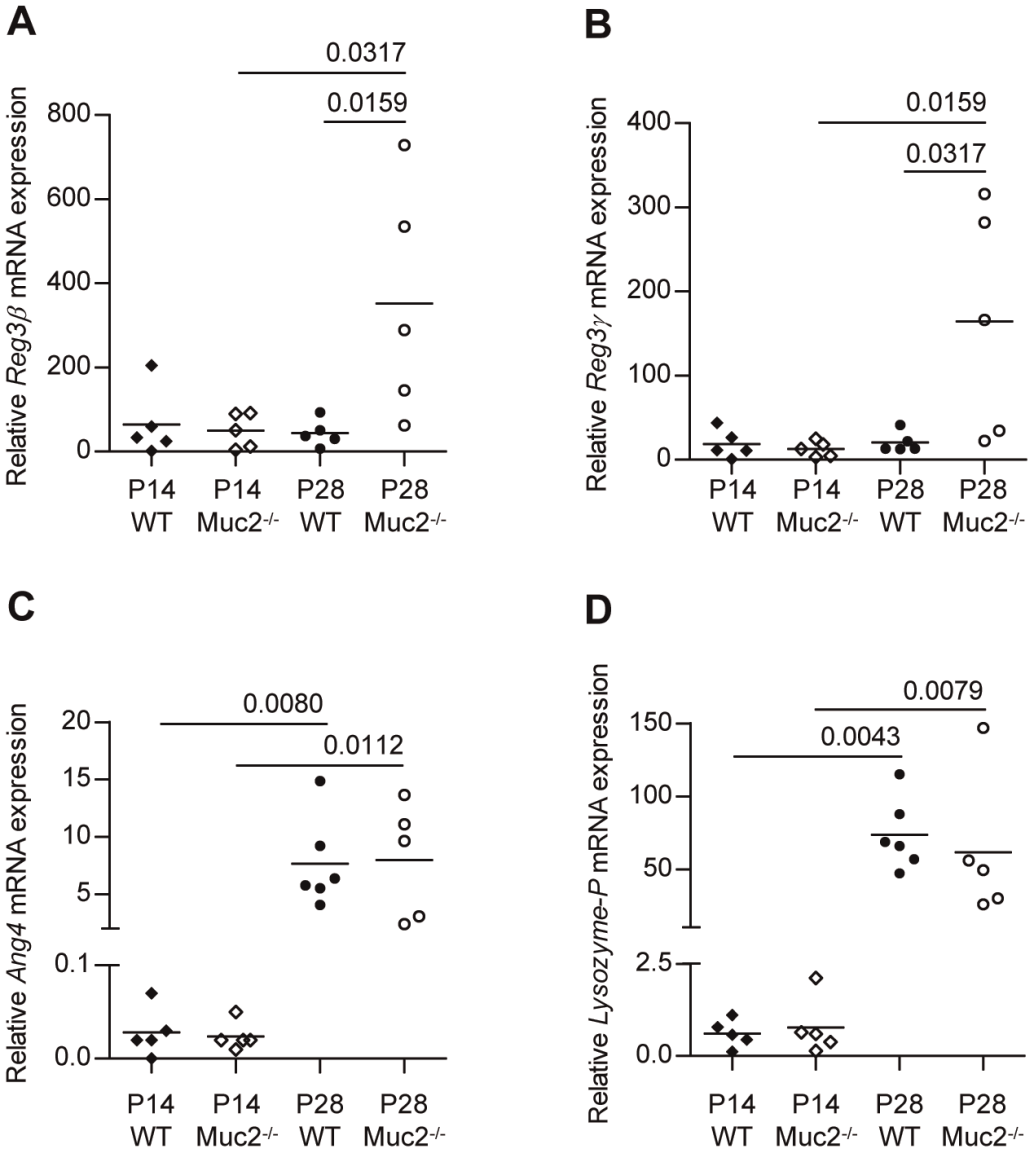
*Reg3β*, *Reg3γ*, *Ang4* and *lysozyme-P* mRNA levels in the jejunum. Small intestinal expression of *Reg3β* (A), *Reg3γ* (B), *Ang4* (C) and *lysozyme-P* (D) mRNA expression. The relative mRNA expression levels were normalized to *Actb* and expressed as median. *P* values are indicated when expression levels between groups differ statistically. Groups are depicted as: WT, P14 ♦; Muc2^−/−^, P14 ◊; WT, P28 •; and Muc2^−/−^, P28 ○.

We next analyzed the mRNA expression levels of Reg3β, Reg3γ, Ang4, and lysozyme-P in the intestinal tissues of WT and Muc2^−/−^ mice. In the jejunum *Reg3β* and *Reg3γ* mRNA levels were comparable between WT and Muc2^−/−^ mice at P14 (Figure 8A and 8B). In contrast, at P28 *Reg3β,* and *Reg3γ* mRNA levels significantly increased in the Muc2^−/−^ mice, whereas these remained stable in the WT mice. The mRNA levels of *Ang4* and *lysozyme-P*, genes which in the small intestine are synthesized by Paneth cells, were comparable between the jejunum of WT and Muc2^−/−^ mice at P14 as well as P28 (Figure 8C and 8D). Remarkably, from P14 to P28 mRNA levels of both *Ang4* and *lysozyme-P* significantly increased in the jejunum of both types of mice.

**Figure 9:**
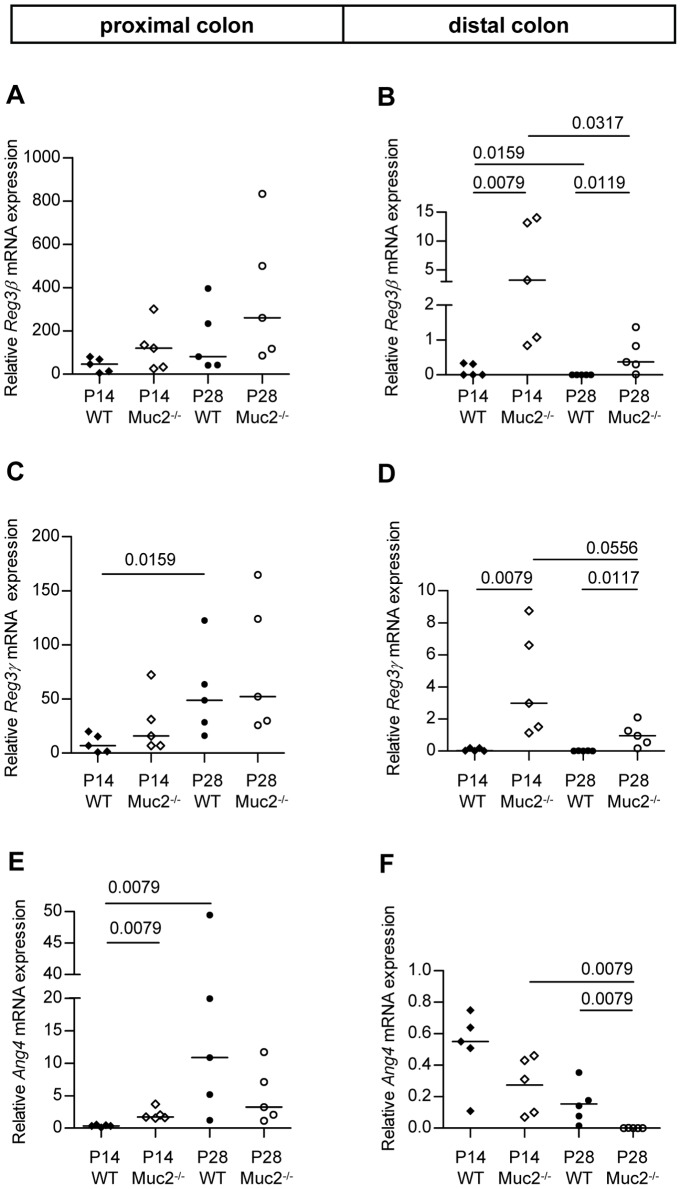
*Reg3β*, *Reg3γ* and *Ang4* mRNA levels in the colon. Expression of *Reg3β* (A,B), *Reg3γ* (C,D), *Ang4* (E,F) mRNA in colonic tissue of WT and Muc2^−/−^. Proximal and distal colonic expression levels are shown in the left and right panels, respectively. The relative mRNA expression levels were normalized to *Actb* and expressed as median. *P* values are indicated when expression levels between groups differ statistically. Groups are depicted as: WT, P14 ♦; Muc2^−/−^, P14 ◊; WT, P28 •; and Muc2^−/−^, P28 ○.

A comparison of *Reg3β*, *Reg3γ* and *Ang4* mRNA levels within colonic tissue of WT mice and Muc2^−/−^ mice revealed six important findings (Figure 9). 1) In WT as well as Muc2^−/−^ mice, higher *Reg3β*, *Reg3γ*, and *Ang4* mRNA levels were found in the proximal colon compared to the distal colon at P14 and P28. 2) During aging from P14 to P28, *Reg3β*, *Reg3γ*, and *Ang4* mRNA levels in the proximal colon increased in WT as well as Muc2^−/−^ mice. 3) Muc2^−/−^ mice had significantly increased *Reg3β* and *Reg3γ* mRNA levels in the distal colon at P14 and P28 compared to WT mice, a similar trend was also observed in the proximal colon. 4) In the distal colon, *Reg3β* and *Reg3γ* mRNA expression levels were hardly detectable in WT mice. 5) *Reg3β*, *Reg3γ*, and *Ang4* mRNA levels in the distal colon of Muc2^−/−^ mice seemed to decrease during aging from P14 to P28. 6) Colonic expression levels of *Reg3β*, *Reg3γ*, and *Ang4* mRNA showed considerable mouse to mouse variation.

### Quantitative Analysis of Reg3β and Reg3γ Protein Levels

Expression of Reg3β and Reg3γ protein was studied by Western-blot analysis. At P14 protein expression levels were below the detection limit in the jejunum of WT as well as Muc2^−/−^ mice (data not shown). At P28, expression of both Reg3β (Figure S4A) and Reg3γ (Figure S4B) was significantly increased in Muc2^−/−^ mice compared to WT mice, in which Reg3β and Reg3γ protein levels remained undetectable. These data correlate with the jejunal Reg3β and Reg3γ mRNA expression levels as presented in Figure 8A and B. Expression of Reg3β and Reg3γ protein in the distal colon was not detectable by Western-blot (results not shown).

## Discussion

In the present study we examined the expression pattern and localization of Reg3β, Reg3γ and Ang4 in intestinal tissue of WT and Muc2^−/−^ mice at the age of 14 days and post weaning at 28 days. We demonstrated that the expression of these genes, which play a role in innate defense and shaping of the bacterial community in the intestine, differs between WT and Muc2^−/−^ mice before and after weaning. These changes might be related to alterations in the composition of the microbiota during this time frame and in Muc2^−/−^ mice also to the altered interactions between bacteria and the intestinal epithelial cells in the absence of the intestinal mucus layer.

Although there were no morphological changes in the small intestine and proximal colon of Muc2^−/−^ mice, these mice did show major differences in the expression pattern and localization of the C-type lectins Reg3β and Reg3γ compared to WT mice. Specifically, in WT mice the Reg3 proteins were only clearly expressed at P14 in the jejunum and ileum. Moreover, expression of the Reg3 proteins was weak or below detection level in this type of mice at P28. In contrast, in Muc2^−/−^ mice these Reg3 proteins were strongly expressed in the entire small intestine from duodenum till ileum at P14 as well as P28. The differences in Reg3 protein expression pattern within WT mice between P14 (i.e., before weaning) and P28 (i.e., after weaning) suggests that bacterial colonization, which is known to change during the weaning period, is highly likely to influence/regulate Reg3β and Reg3γ expression, which is supported by the findings of other authors [18], [19]. Along the same line, differences in Reg3 protein expression between WT mice and Muc2^−/−^ mice might point to a difference in the composition of the microbiota between these mice and thus that mucins, in particular mucin Muc2, also influence the composition of the microbiota.

It was previously shown that Reg3γ and Ang4 are expressed in small intestine and localized in Paneth cells [19], [22] and *Reg3β* mRNA was shown in colonic goblet cells and columnar cells [18]. Our immunohistochemical analysis revealed that in the small intestine of WT mice and Muc2^−/−^ mice Reg3γ and Ang4 are indeed expressed by Paneth cells. Yet, in Muc2^−/−^ mice Paneth cells also express Reg3β. These data suggest that in the absence of Muc2, and thus in the absence of a mucus layer, Paneth cells increase their innate defense capacity by expressing Reg3β. Even more importantly, we also demonstrated that during initial colonization Reg3β and Reg3γ are not exclusively expressed by Paneth cells, but also by enterocytes and goblet cells in the small intestine and proximal colon of WT and Muc2^−/−^ mice. Additionally, goblet cells in the proximal colon also appeared to synthesize Ang4. Besides secreting Relmβ, which is suggested to have an immune effector function [31], [32], [33], goblet cells were until now not known to play a role in innate defense responses via the secretion of bactericidal proteins. Our demonstration that Reg3β, Reg3γ, and Ang4 are expressed by goblet cells highlights a new and important role for goblet cells in innate defense and in helping to shape the bacterial community. Overall, the spatial Reg3β and Reg3γ expression is remarkable in the sense that these proteins are expressed in at least 3 different epithelial cell lineages within the intestine, namely goblet cells, enterocytes, and Paneth cells.

Focusing on Reg3 expression levels, we demonstrate that small intestinal expression of *Reg3β* and *Reg3γ* mRNAs and proteins were increased in Muc2^−/−^ mice compared to WT mice at P28 just after weaning. These data imply that loss of a protective mucus layer as in Muc2^−/−^ mice leads to an increased innate defense response, probably as a result of increased epithelial-bacterial interactions and altered bacterial colonization as weaning is known to alter the composition of the microbiota. Small intestinal expression levels of *Ang4* mRNA were also increased after weaning in WT as well as Muc2^−/−^ mice. We additionally showed that expression levels of Ang4 in the small intestine resemble lysozyme-P levels over time. As it is known that Paneth cell development occurs after birth in mice, with a complete constitution of the Paneth-cell lineage from the age of 3 to 4 weeks [34], the increased expression of *lysozyme-P* and *Ang4* mRNAs at P28 compared to P14 is most likely due to increased Paneth cell numbers during development from P14 to P28.

Expression levels of *Reg3β*, *Reg3γ*, and *Ang4* mRNA in the proximal colon were considerably higher compared to expression levels in the distal colon of WT and Muc2^−/−^ mice at both time points investigated. An explanation for this could be that an intrinsic program encoded in the epithelial cells controls the segmental expression of the studied innate defense molecules. On the other hand, altered expression of the *Reg3* and *Ang4* genes might be related to changes in the composition of the microbiota as demonstrated for *Reg3γ* in a simplified model where germ-free mice are sequentially colonized with *Bacteroides thetaiotaomicron* and then *Bifidobacterium longum* [35]. Specifically, colonization of germ-free mice with *Bacteroides thetaiotaomicron* induced *Reg3γ* expression, but this was lowered by the subsequent introduction of *Bifidobacterium longum.* Thus the observed differences in *Reg3β*, *Reg3γ*, and *Ang4* gene expression levels between the proximal and distal colon might be related to differences in the composition of the microbiota in these parts of the intestine. Indeed, it has been shown that the composition of mucosa-associated bacterial species may differ up to 4% between the right colon (i.e. proximal colon) and left colon (i.e. distal colon) [36], [37].

Regardless of the mechanisms of *Reg3* gene and *Ang-4* gene regulation, there seems to be an inverse correlation between *Reg3β*, *Reg3γ*, and *Ang4* gene expression levels and the location of colitis in Muc2^−/−^ mice. Namely, morphological signs of colitis are only observed in the distal colon of Muc2^−/−^ mice at P28, where and when the expression levels of Reg3β, Reg3γ, and Ang4 were the lowest, but not in the proximal colon, where Reg3β, Reg3γ, and Ang4 levels were the highest. These findings could imply that Reg3 proteins and/or Ang4 regulate intestinal inflammation directly or indirectly. Interestingly, studies from Folch-Puy et al. indicate that PAP-I (also known as HIP, p23, or Reg2 protein) directly inhibits the inflammatory response by blocking NF-κB activation through a STAT3-dependent mechanism [38]. When Reg3 proteins and/or Ang4 indeed limit intestinal inflammation one might even speculate that the distal colon is more prone to develop colitis than the proximal colon because *Reg3β*, *Reg3γ*, and *Ang4* expression levels are lower in the distal colon than in the proximal colon. However, the inflammatory modulating capacities of the Reg3 and Ang4 proteins still remain to be proven.

Expression levels of *Reg3β* and *Reg3γ* mRNAs were consistently higher in Muc2^−/−^ mice compared to WT mice in both the proximal and distal colon. This is most likely due to increased commensal bacterial-epithelial interactions in Muc2^−/−^ mice compared to WT mice, which has been demonstrated before [1]. Given that the glycans on mucins are a nutrient source for bacteria [39], loss of Muc2 is likely to influence the composition of the microbiota. Differences in the composition of the colonic microbiota between WT and Muc2^−/−^ mice might in their turn also influence Reg3 and Ang4 protein expression.

After weaning, expression levels of *Reg3β*, *Reg3γ*, and *Ang4* mRNAs in the proximal colon were increased in WT and Muc2^−/−^ mice. Cash et al. showed that *Reg3γ* mRNA expression increased during the weaning period in the small intestine of conventionally raised mice, but not in germ-free mice [19]. The same accounts for Ang4 expression in the small intestine [22]. It is known that the density and complexity of the microbiota increases significantly after weaning. This can be explained by the food source itself, serving as a substrate for specific bacteria, but also by the loss of protective factors that are present in mother’s milk but not in plant-based chow *e.g.* sCD14, sTLR2, TGFβ, IL-10, and lactoferrin [40], [41], [42], [43]. Anyway, increased expression of Reg3β and Reg3γ at P28 in WT as well as Muc2^−/−^ mice might be regarded as an innate response to alterations in the number and composition of the microbiota that are related to the weaning process.

In summary, this study demonstrates that Reg3β and Reg3γ can be expressed in diverging cell lineages, namely enterocytes, Paneth cells, and goblet cells. This study also highlights a new role for goblet cells in host innate immunity by demonstrating that they can produce the bactericidal peptides Reg3β, Reg3γ, and Ang4. Additionally, absence of Muc2 resulted in strong up-regulation of *Reg3β* and *Reg3γ* mRNAs in the small intestine and colon, suggesting altered bacterial-epithelial signaling in Muc2^−/−^ mice, leading to increased innate defense capacity. Alterations in *Reg3* and *Ang4* gene expression were related to weaning from mother’s milk, which is known to alter the composition of the microbiota. Therefore an important role for bacteria in regulation of *Reg3* and *Ang4* gene expression is suggested. Furthermore, morphological signs of colitis were observed in the distal colon of Muc2^−/−^ mice, where expression levels of *Reg3β*, *Reg3γ*, and *Ang4* mRNAs were the lowest, but not in the proximal colon where expression levels of these genes were the highest. These findings might point toward a role for Reg3 proteins and/or Ang4 in regulating intestinal inflammation.

## Supporting Information

Figure S1 **Morphology of the jejunum, proximal and distal colon.** H&E staining of jejunum (A, B, G, H), proximal colon (C, D, I, J) and distal colon (E, F, K, L) of WT mice (A, C, E, G, I, K) and Muc2^−/−^ mice (B, D, F, H, J, L) at P14 (A–F) and P28 (G–L). Bar represents 150 µm.
(TIF)

Figure S2 **Goblet cell-specific expression of Reg3β and Reg3γ in the jejunum of WT mice.** Serial sections were stained for Muc4 (A) Reg3β (B), Reg3γ (C) and periodic acid-Schiff’s (PAS) (D). Co-localization of Reg3β and Muc4 is shown by white arrowheads and co-localization of Reg3γ and PAS staining is shown by black arrowheads. Bar represents 75 µm.
(TIF)

Figure S3 ***Ang4*** **mRNA expression by jejunal enterocytes and Paneth cells.** Localization of *Ang4* mRNA at P14 in small intestine of Muc2^−/−^ mice. Arrowheads indicate *Ang4* mRNA-positive Paneth cells.
(TIF)

Figure S4 **Reg3β and Reg3γ protein levels in the small intestine.** Small intestinal expression of Reg3β (A) and Reg3γ (B) protein. Photomicrographs depict representative examples of Reg3β and Reg3γ expression in WT and Muc2^−/−^ mice, and corresponding β-Actin expression. The relative protein levels were normalized to β-Actin and expressed as median. *P* values are indicated when expression levels between groups differ statistically. Groups are depicted as: WT, P28 •; and Muc2^−/−^, P28 ○.
(TIF)
