# Supplementary figures and images for: Mucin Muc2 Deficiency and Weaning Influences the Expression of the Innate Defense Genes Reg3β, Reg3γ and Angiogenin-4
Source: PLoS One. 2012 Jun 19;7(6):e38798. doi: 10.1371/journal.pone.0038798 (PMC3378615; doi:10.1371/journal.pone.0038798)

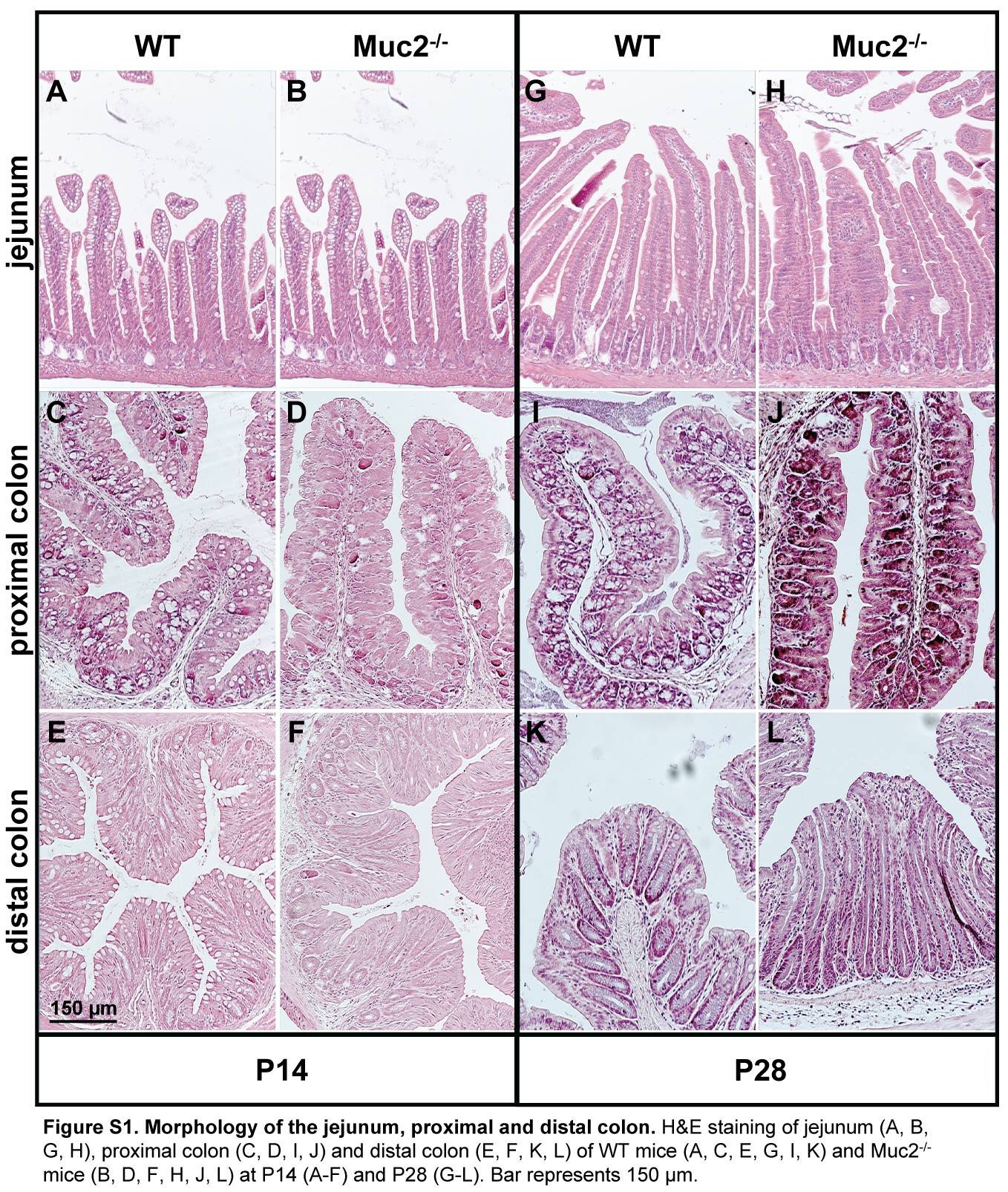

Supplement: Figure S1 — Morphology of the jejunum, proximal and distal colon. H&E staining of jejunum (A, B, G, H), proximal colon (C, D, I, J) and distal colon (E, F, K, L) of WT mice (A, C, E, G, I, K) and Muc2−/− mice (B, D, F, H, J, L) at P14 (A–F) and P28 (G–L). Bar represents 150 µm. (TIF) [file pone.0038798.s001.tif]

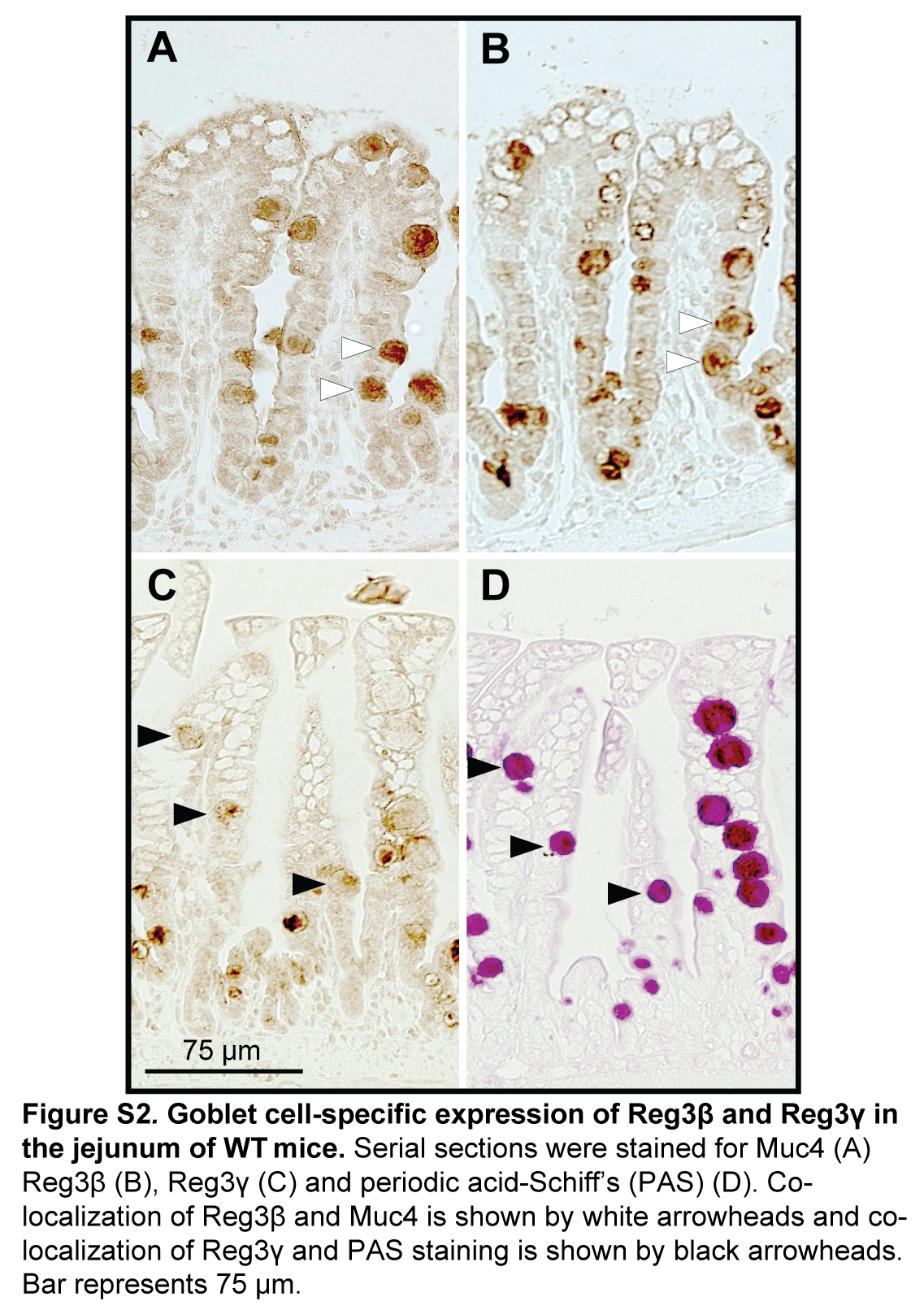

Supplement: Figure S2 — Goblet cell-specific expression of Reg3β and Reg3γ in the jejunum of WT mice. Serial sections were stained for Muc4 (A) Reg3β (B), Reg3γ (C) and periodic acid-Schiff’s (PAS) (D). Co-localization of Reg3β and Muc4 is shown by white arrowheads and co-localization of Reg3γ and PAS staining is shown by black arrowheads. Bar represents 75 µm. (TIF) [file pone.0038798.s002.tif]

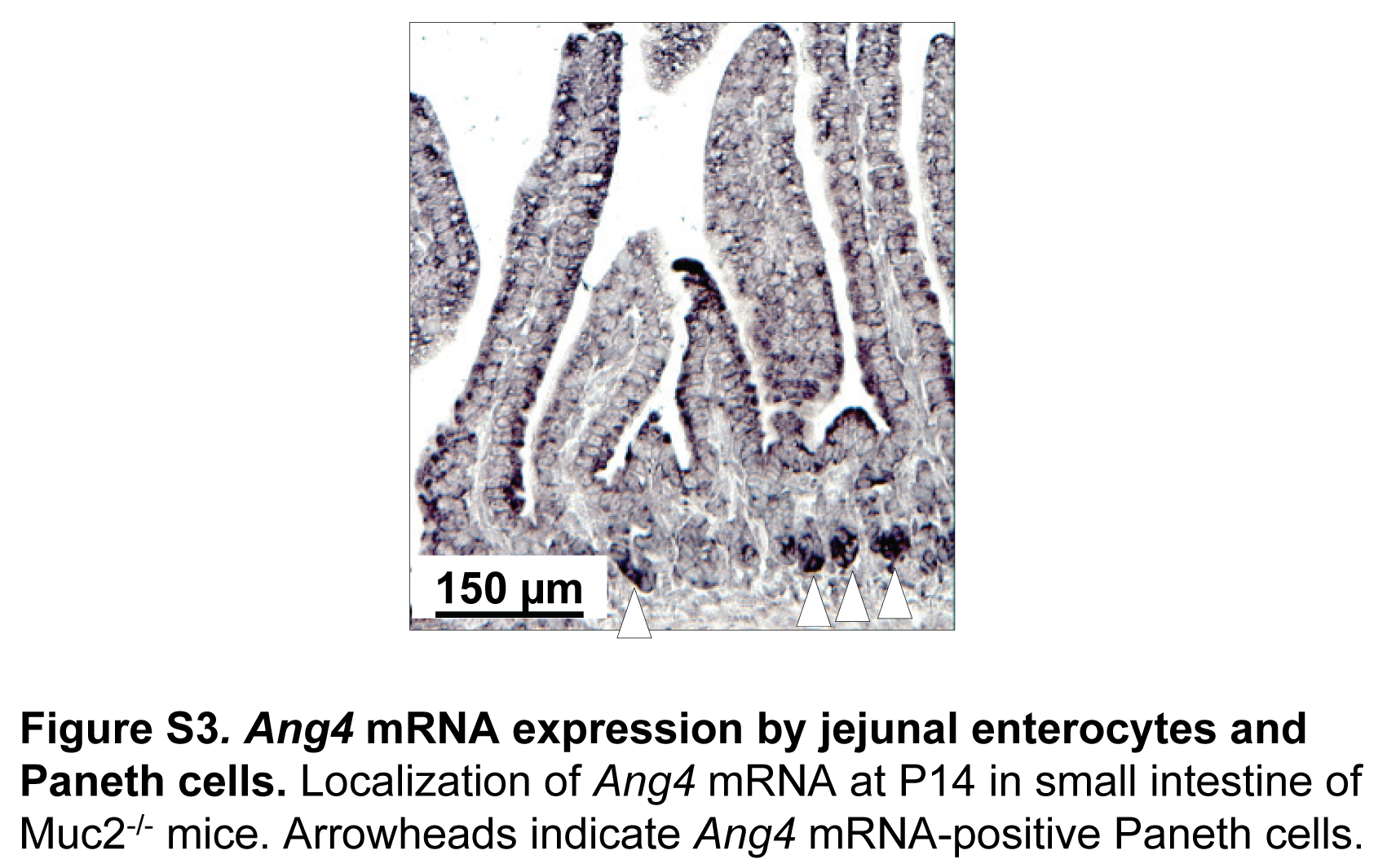

Supplement: Figure S3 — Ang4 mRNA expression by jejunal enterocytes and Paneth cells. Localization of Ang4 mRNA at P14 in small intestine of Muc2−/− mice. Arrowheads indicate Ang4 mRNA-positive Paneth cells. (TIF) [file pone.0038798.s003.tif]

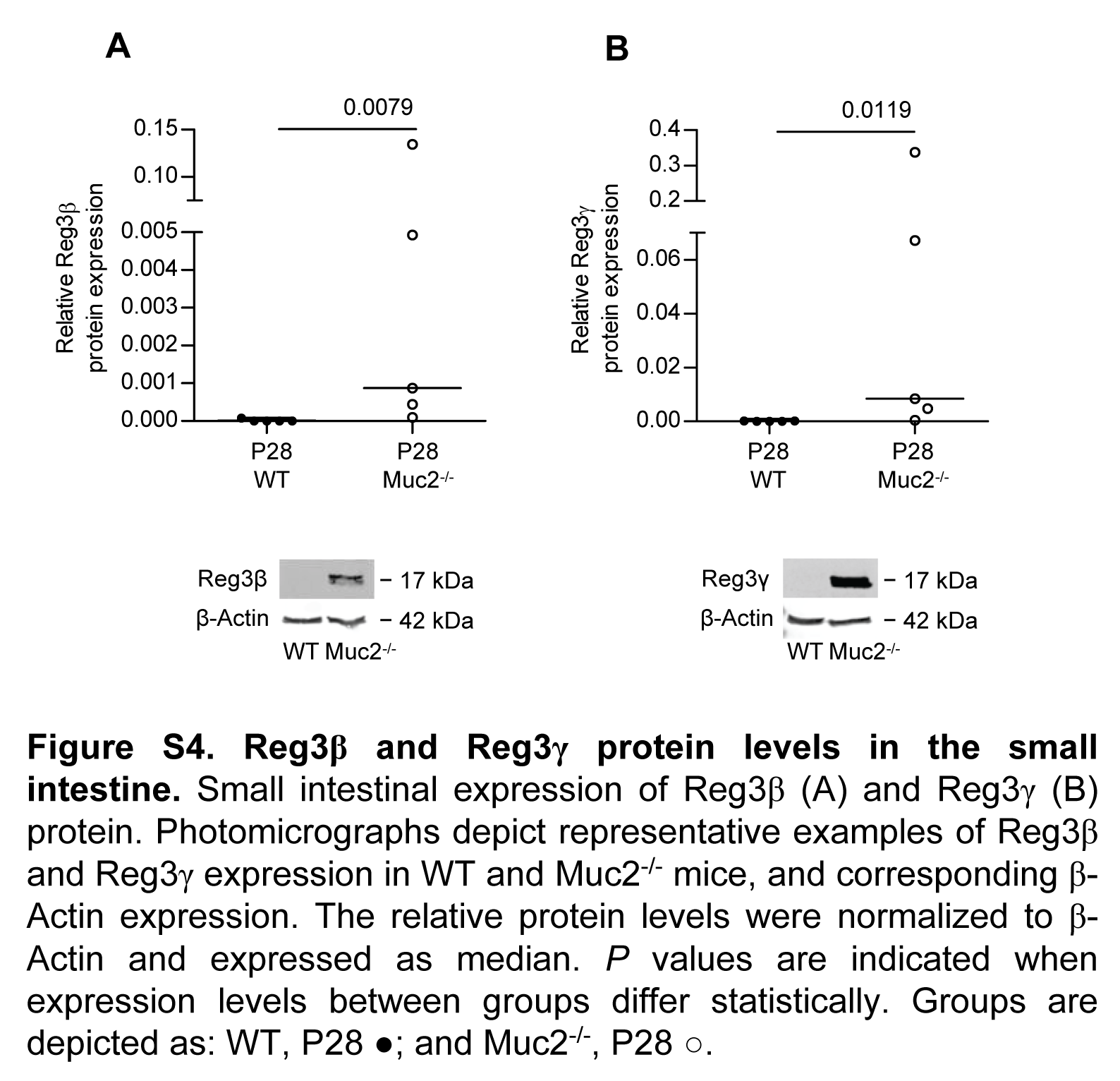

Supplement: Figure S4 — Reg3β and Reg3γ protein levels in the small intestine. Small intestinal expression of Reg3β (A) and Reg3γ (B) protein. Photomicrographs depict representative examples of Reg3β and Reg3γ expression in WT and Muc2−/− mice, and corresponding β-Actin expression. The relative protein levels were normalized to β-Actin and expressed as median. P values are indicated when expression levels between groups differ statistically. Groups are depicted as: WT, P28 •; and Muc2−/−, P28 ○. (TIF) [file pone.0038798.s004.tif]
